# Shoulder Joint Dislocation as an Unusual Complication of Defibrillation Threshold Testing Following Subcutaneous Implantable Cardioverter-Defibrillator Implantation

**DOI:** 10.1016/s0972-6292(16)30818-x

**Published:** 2014-12-15

**Authors:** Amit Noheria, Yong-Mei Cha, Samuel J Asirvatham, Paul A Friedman

**Affiliations:** Division of Cardiovascular Diseases, Mayo Clinic, Rochester, MN

**Keywords:** Subcutaneous implantable cardioverter-defibrillator, defibrillation threshold testing, shoulder dislocation, complication, device implantation

## Abstract

A 53-year-old man underwent implantation of a totally subcutaneous ICD (S-ICD; Boston Scientific). He was positioned supine, with the left arm abducted, externally rotated (i.e. palm up) and strapped to the arm extender. The generator was placed in the left mid-axillary line along the 5th-6th intercostal spaces and the defibrillation coil was tunneled anterior to the sternum. Defibrillation threshold (DFT) testing with 65 Jcaused a forceful pectoralis twitch. The patient woke up with a painful anteriorly dislocated left shoulder. Glenohumeral dislocation due to DFT testing has not been previously reported. It is likely that this complication is specific to the S-ICD implantation, and is related to positioning with the arm abducted, externally rotated, and immobilized, and use of greater defibrillation energy with current pathway through the bulk of the pectoralis muscle.Precautions may include extending the arm palm down, strapping the arm loosely, and adduction of the arm for DFT testing.

## Case Report

A 53-year-old man with hypertension, obesity (BMI 31.4 kg/m2), remote history of traumatic left shoulder dislocation,and hypertrophic cardiomyopathy with sudden death risk factors of ventricular septal thickness 35 mm, non-sustained ventricular tachycardia, exercise induced drop in blood pressure, and myocardial scar on magnetic resonance imaging, underwent implantation of a totally subcutaneous ICD (S-ICD; Boston Scientific). Propofol, ketamine, fentanyl and midazolam were used for sedation. He was positioned supine, with the left arm abducted, externally rotated (i.e. palm up) and strapped to the arm extender. The generator was placed in a subcutaneous pocket in the mid-axillary line along the 5thto 6th intercostal spaces. The lead was tunneled under the skin to a 2 cm incision just above the xiphisternum, and the coil electrode was further tunneled anterior to the sternum. Fifty Hz stimulation caused vigorous left pectoralis contractions during induction of ventricular fibrillation (VF). The device appropriately detected VF and successfully shocked with 65 J (maximum 80 J), also causing a forceful pectoralis twitch. Thirty seconds of post shock pacing caused lesser degree of muscle twitching.

**Figure 1:**
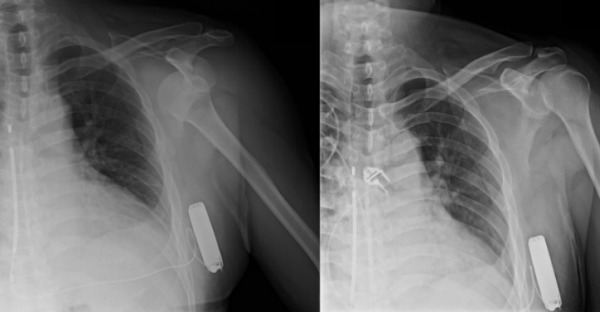
Radiographs showing (left panel) anterior glenohumeral dislocation and (right panel) return of the humeral head to the glenoid cavity after closed reduction. Also seen is the S-ICD system.

Following the procedure the patient complained of significant pain in the left shoulder that was not responsive to ketorolac, fentanyl and midazolam. On examination there was a gross deformity of the shoulder with the humeral head palpable anteriorly. He had intact upper extremity pulses and sensory exam, and normal motor function of the elbow, wrist and digit flexors and extensors. (Figure 1A) After re-sedating the patient, the shoulder was closed-reduced back to the glenoid cavity with gentle traction and forward elevation. (Figure 1B) The discomfort immediately resolved and a shoulder sling was prescribed for 2-3 weeks.

## Discussion

The S-ICD has been shown to be a safe and effective alternative to transvenous lead placement for patients without pacing indications, with excellent sensing and defibrillation of VF. [1,2] The S-ICD eliminates/reduces the risks of tricuspid valve damage, blood-stream infections, thromboembolic complications and need for endovascular lead extractions, and is presumably a more durable system. Limitations include inappropriate therapies due to T-wave oversensing and inability to provide anti-tachycardia pacing. [1]

**Figure 2:**
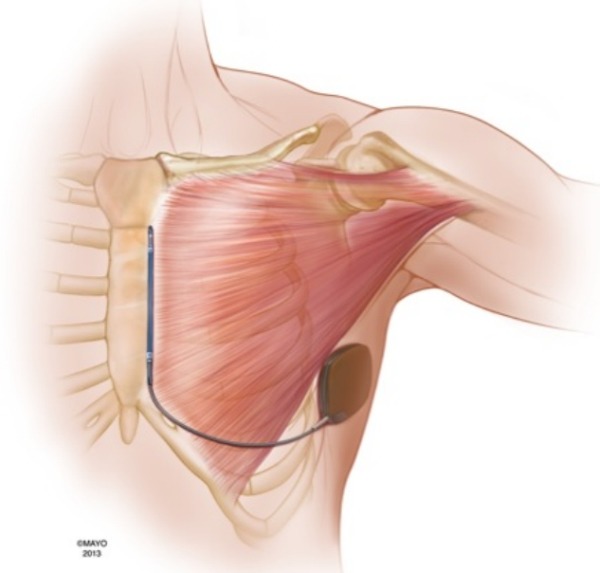
Illustration showing the pectoralis major flanked by the S-ICD system, with the generator inferoposterior to the lateral margin, and the shock coil just medial to the sternal insertion. The shocking vector between the generator and the coil electrode captures the bulk of the pectoralis major. The contraction of the pectoralis will pull the humerus head anteriorly and inferiorly, with a tendency to pivot out of the glenoid cavity if the forearm is strapped and immobilized.

Humeral dislocation commonly occurs in context of high-energy injuries.[3] The violent external rotation and abduction of the arm causes the humeral head to dislocate anteriorly. Shoulder dislocation related to surgical positioning or defibrillation threshold testing (DFT) has not been reported, though one dislocation with external cardioversion has been described.[4] Fortransvenous ICD implantation, the patient is strapped as a single unit, with the arms on the side free to move along with the torso. However, to place the S-ICD generator in the left mid-axillary line, the left arm is abducted, externally rotated and strapped to the arm extender. With electrical capture of the pectoralis major that would normally cause abduction and internal rotation of the humerus, the humeral head is at a risk for anterior dislocation because the arm is immobilized. (Figure 2)

To our knowledge this is the first report of glenohumeral dislocation due to DFT testing. It is likely that this rare complication is specific to the S-ICD implantation, and is related to positioning with the arm abducted, externally rotated, and immobilized. Greater defibrillation energy, prolonged stimulation during VF induction (3-5 seconds), and current pathway through the bulk of the muscle (Figure 2) may lead to vigorous pectoralis contraction. Precautions should be utilized, especially for patients with prior shoulder instability or strong upper extremity musculature. Strategies may include extending the arm palm down, strapping the arm loosely, and adduction of the arm for DFT testing, although this may risk the sterility of the field. Immediate identification of shoulder dislocation during the procedure and closed reduction before the patient is awakened from anesthesia can alleviate the associated pain.
